# Interpersonal Dynamics: 1v1 Sub-Phase at Sub-18 Football Players

**DOI:** 10.2478/hukin-2013-0018

**Published:** 2013-03-28

**Authors:** Filipe Manuel Clemente, Micael Santos Couceiro, Fernando Manuel Lourenço Martins, Gonçalo Dias, Rui Mendes

**Affiliations:** 1 Faculty of Sport Sciences and Physical Education – University of Coimbra, Portugal.; 2 RoboCorp, Coimbra Institute of Engineering – Polytechnic Institute of Coimbra, Portugal.; 3 RoboCorp, Coimbra College of Education – Polytechnic Institute of Coimbra, Portugal.; 4 Instituto de Telecomunicações, Delegação da Covilhã, Portugal (IT).; 5 Interdisciplinary Centre for the Study of Human Performance – Lisbon Technical University, Portugal.

**Keywords:** sport analysis, constraints, instruction, interpersonal dynamics, football

## Abstract

The performance of football players within game context can be analyzed based on their ability to break or (re)balance the attacker-defender dyad. In this context, the analysis of each sub-phase (e.g., 1v1, 2v2) presents a feature that needs to be taken into account in sports analysis. This study aims to investigate the interpersonal dynamics dyad formed by the attacker and the defender in 1v1 situations with a goalkeeper. A sample of 11 football male players (age: 17.91 ± 1.04 years) with 8.6 ± 1.52 years of practice experience participated in the study. Analyzing the 1v1 sub-phase, results show that the distance, speed and angular amplitude between the attacker and the defender increases, especially when the attacker attempts to overtake the defender (i.e., score a goal). We conclude that decision-making emerges from the perception that players draw from the action, actively and consistently interacting to find solutions to emerging problems within the game context.

## Introduction

Team sports games can be characterized as a dynamic system that includes standards with great variability and complexity (Davids et al., 2005). Team ball sports are dynamic systems that present highly complex phenomena, since the number of degrees-of-freedom (*dof*) that characterize the relationships between the players and the environment evolve over time (Passos et al., 2006).

From 1-vs-1 to many-vs-many, the interactions in sports context may be analyzed based on the attacker-defender symmetry. Therefore, the attacker seeks to break the symmetry with the defender (i.e., equilibrium between attacker and defender where the defender remains in the defensive position considering the goal), while the defender tries to maintain the symmetry with the attacker. Thus, sports might be considered as dynamical systems that lie between the boundaries of stability and instability (McGarry, 2005).

Consequently, interpersonal coordination within teams emerges from the coupling between players and the context (Passos et al., 2006). Based on these insights, the behaviour of interacting players in team games could be interpreted as an emergent process resulting from the spatiotemporal relations established during the competitive performance sub-phases (Duarte et al., 2012). Thus, the sub-phases include interpersonal patterns that result from the dyad formed between attacker(s)-defender(s) (Davids et al., 2005). Based on McGarry and Franks (2007) work, dyad synergies (i.e., permanent interactions over time) as attacker-defender interactions, may exhibit non-linear properties, including the sustained periodic behaviour, and specific types of interpersonal coordination may emerge under the influence of environment, personal, and task constraints (*e.g.*, specific field markings and rules of the game).

Recent studies in basketball (Davids et al., 2006), rugby (Passos et al., 2006) and football (Duarte et al., 2010; Duarte et al., 2012) show that players inter-relate through perception-action couplings, under the influence of various constraints that may arise due to tactical positioning, strategy and game objectives (Duarte et al., 2010; Duarte et al., 2012). Thus, comparing one player in two different teams or two different situations will not yield the same results. The essence of style of play depends on the players specific properties (Deleplace, 1995; Wade, 1970). Thus, the sum of players that constitutes the team has a preponderant influence in the player’s performance and the evolution of play style (Gréhaigne et al., 2005).

In basketball, research supports that symmetry-breaking processes take place in successful dribbling when abrupt changes in the shape of the curve describing the interpersonal distance of each attacker–defender dyad occurs (Passos et al., 2006).

In rugby, it is suggested that interpersonal distance (*i.e.*, distance between members of the dyad) as a control parameter *per se* cannot explain the emergence of dyadic outcomes. Thus, under the task constraints of rugby union, interpersonal distance can act as an initial potential control parameter that moves the dyadic attacker-defender system towards a region of self-organizing, *i.e.*, the organization of the play is directly influenced by the context (Duarte et al., 2012).

In football, the analysis of phase transitions show that this instant is related to lower values of interpersonal distance and higher values of relative velocity (Duarte et al., 2010). When these control parameters were expressed as a single variable, maximum peaks were related to the exact moment of phase transition. The findings of this research, similarly as the findings of the rugby study, suggested that dyadic system behaviours emerge in an environmental exploratory process that cannot be explained exclusively by only one control parameter (i.e., the player with ball possession try to act in order to perceive new information and improve the action success) (Duarte et al., 2010).

Researchers such as McGarry et al. (2002) described that the quality of the attacker and the defender can be seen by their ability to break or (re)balance the system, i.e., the attacker-defender dyad. Despite this important relationship, few studies have focused their analysis on the 1v1 sub-phase (Duarte et al., 2012). However, research from Davids (2006), Duarte et al. (2010), which analyzed the 1v1 sub-phases (*i.e.*, specific phase of the game), demonstrates that the equilibrium or rupture of the couple depends, among other factors, on the interpersonal distance and relative speed between players.

The attacker-defender dynamics emerge from the perception-action cycles, i.e., a player needs to systematically act in order to perceive new possible actions. The dynamics of the perception, action, and cognition can be reported at two levels of analysis (Davids et al., 2006). The first level characterizes agent–environment interactions, with performer actions detecting information. Reciprocally, this information is used to regulate further actions according to control parameters (Warren, 1998). At this level, the problem is to identify the informational variables that are used to guide behaviour and regulate their actions (Warren et al., 2001). In fact, the coach can help the player to find the best information within the environment, especially using task constraints. These task constraints (*e.g.*, number of players by exercise, the field space, the type of feedback) can help the player to find relevant sources of information. Therefore, the selection process of the task constraints is a key factor that determines the quality and efficacy of the teacher and coaches’ intervention (Chow et al., 2006).

Task constraints can restrict or enable multiple behaviors that players can adopt during a practical situation (Davids et al., 2008). More specifically, task constraints can help the players to center the perception within specific information (Tan et al., 2011). Therefore, they include the rules that constrain spatial and temporal patterns of the movement dynamics during an activity that may be open to interpretation (Handford et al., 1997). It should be noted that individuals concerned with the acquisition of skills should consider the manipulation of significant constraints to achieve a desired response. For instance, a football coach may guide the search for a solution by imposing a rule constraint that only allows shots from outside the penalty area (Handford et al., 1997). Hence, the coach can take use of constraints to forecast the session, thus directing players’ perception to the information considered to be relevant. One of these constraints can be the coach’s instruction.

A study with basketball players by Davids (2006) concluded that the type of instruction influenced the system to behave with a higher instability and, consequently, with a higher diversity of coordination states. Considering that coaches’ information is a trivial action provided over the training session, it may be important to understand how different types of information can constrain players’ behaviour.

Given the above, this study intends to analyze the dynamics of the interpersonal dyad formed by the attacker and the defender in 1v1 football situations with goalkeeper. During the experimental scenario, the coach provides three different types of information to the attacker in order to understand if the verbal information constrain and change the individual behaviour. Therefore, a set of individual metrics that allows verifying the attacker’s behaviour in the 1v1 subphase, such as the interpersonal distance, the velocity, the angular position of the attacker in relation to the defender and the trajectories on the field, will be analyzed.

## Methods

### Participants

This study analyzed 11 male football players of federated teams from Coimbra, Portugal, 17.91 ± 1.04 years of age, and with 8.6 ± 1.52 years of practice experience. All individuals signed the *Free and Clarified Consent Form* respecting the *Helsinki Declaration*. The participants did not have any kind of physical or psychological diseases.

#### Task

The task consisted of the realization of a football offensive attempt (i.e., trying to score a goal) by an attacker in a 1v1 situation plus goalkeeper.

#### Experimental Design

**Figure 1:**
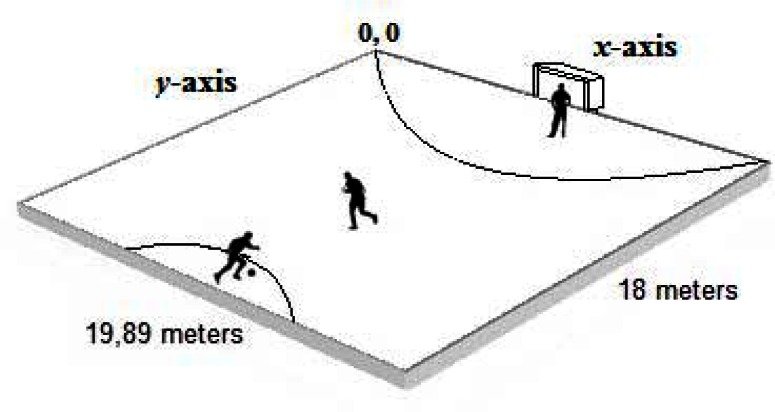
Experimental device and initial positioning of players on the game space.

A football game sub-phase (i.e., 1v1 plus goalkeeper) was implemented in a scenario of 19.6 m wide by 18 m long, with markers at the edges of the field (Figure 1).

At the beginning of each trial, the defender was located 6 m away from the attacker, thus ensuring an initial distance that would enable the attacker to choose the first action. The defender was positioned in the center of the field, 18 m away from the goal (the midfield line). The goalkeeper was restricted to his goal line.

Ten trials were performed in each practice condition (conservative, risk and neutral), i.e., each offensive player performed a total of 30 trials.

During these 30 trials, the opponent defender and goalkeeper were the same. Nevertheless, each time the attacker changed, the remaining players (defensive players) also changed to reduce the possibility of identifying patterns in their behaviour. Prior to the first study, an offensive solution was provided to each participant.

In each practice condition, the attacker was told to implement the test using an offensive strike (i.e., score a goal). On the other hand, the defender was reported to prevent the goal. Before the start of each trial, the instructional constraint was provided (i.e., conservative, risk or neutral), and the attacker began his offensive attempt. Between each offensive attempt, players passively recovered for 30 s. All trials followed the rules of organized football for this age group.

### Task Conditions

Participants interacted in 1v1 situations with goalkeepers. Each participant performed the task under the influence of three types of instructional constraints: 1) conservative 2) risk and 3) neutral. In the conservative instruction, the attacker was informed that his team was winning and thus to attack whenever he had the opportunity. In the risky instruction, the attacker was informed that the game would soon end and that his team was losing thus resulting in a risky behaviour. Finally, in the neutral instruction, it was reported that the attacker should try to score a goal.

### Apparatus

The players’ actions were captured using a digital SLR camera (Canon EOS 500D) with a capacity to process images at 30 Hz (i.e, 30 frames per second). The camera was placed 4.53 m above ground to capture the whole task. Official football balls were used for this specific age group of players (65 cm radius and weight of 380 grams). Orange and yellow vests were used by the attacker and defender, respectively. Players’ trajectories were analyzed using MATLAB. The software is a programming environment for algorithm development, data analysis, visualization, and numerical computation.

### Procedures

For spatial calibration, 27 points in the field margins were used. After capturing the football offensive attempts, the physical space was calibrated using direct linear transformation (i.e., DLT), which relates the objects’ position (i.e., players) in the metric space with the corresponding object in the image (Duarte et al., 2010).

After calibration, we proceeded to the manual tracking of players at regular intervals of 0.12 s, resulting in the Cartesian positioning of each player. After obtaining each player’s trajectory in the *x*, *y* plane (i.e., plane defined by the field), we obtained the distance between players and the goal using the following equation:

(1) $$d=\sqrt{x^{2}+y^{2}}$$

Higher values of velocities were identified as critical to the success of the attacker or the rebalancing of the dyad by the defender opponent (Duarte et al., 2010). Thus, the players’ speed throughout each offensive attempt was calculated using the following equation:

(2) $$v=\sqrt{v_{x}^{2}+v_{y}^{2},}$$

wherein *v_x_* and *v_y_* respectively corresponds to the discrete derivative of the position in the *x*-axis and the *y*-axis over time, calculated as:

(3) $$\{\begin{array}{l} v_{x}=\frac{x(t)-x(t-1)}{\Delta t}\\ v_{y}=\frac{y(t)-y{\left(t-1\right)}^{\prime }}{\Delta t} \end{array}$$

where Δ*t* represents the discrete time interval which corresponds to 0.12 s. This time interval assured the manual tracking’ efficiency, thus allowing identifying players’ trajectories and reducing the size of the raw data.

In order to understand the relationship between the position of the attacker and the defender, we calculated the angular positioning between them. To that end, we considered an angle of 0° as the angle between the defender and the attacker when they form a line perpendicular to the goal, being the defender closer to it.

The way the angle increases or decreases follows the basic principles of the unit circle where the origin of the referential is the defender. This means that when the attacker overtakes the defender, i.e., when the attacker is closer to the goal, the angle will be situated on the 2nd or 3rd quadrant, i.e., 90º<|θ|≤180°.

**Figure 3:**
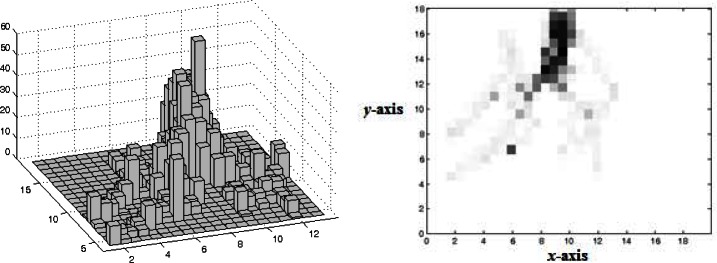
Illustrative image of a histogram (left) and its heat map (right) representative of the most occupied zones of the field by a player in a practice condition

The mapping allowed the construction of frequency histograms based on the spatial distribution of the attacker. Each heat map refers to a condition of practice for each player, i.e., each heat map represents 10 trials of each player in each condition of practice. For this purpose, the whole scene was split in a 20 × 20 matrix resulting in a resolution lower than 1 m^2^, thus obtaining a histogram representative of the most occupied zones of the field by a given player in a given practice condition. Figure 3 (left) illustrates an example of an obtained histogram.

To support the analysis of the occupied zones we proceeded to the design of heat maps (Figure 3 right). These heat maps consist of a graphical representation of the data in which the frequency values obtained by the spatial distribution histograms are represented in a two-dimensional table with different colors. The darker colors represent a higher occupation frequency in a certain zone of the field. This graphical representation allows a quick view of the data, giving a potential to analyze possible trends of spatial occupation of attackers.

In addition, to analyze the traveled distance, we also proceeded to the statistical analysis of required time the attackers needs to complete the offensive attempt in each practice condition. This analysis allows to verify if different instructions provided by the coach results in differences while achieving the task. Thus, in each trial, the time spent by the attacker to complete the offensive process was also collected.

The one-way ANOVA was used to establish the statistically significant differences between football players, in each practice condition. The assumption of normality distribution of one-way ANOVA in the three practice conditions (i.e., conservative, neutral and risk) was investigated using the Kolmogorov-Smirnov test with correction Lillefors. It was found that the distributions are not normal in the dependent variable. Although it was not normal, since n = 110, using the Central Limit Theorem we assumed the assumption of normality (Akritas and Papadatos, 2004). The analysis of homogeneity was carried out using the Levene test. It was found that there is no uniformity of practice under the previously mentioned conditions. However, despite the lack of homogeneity, the F test (ANOVA) is robust to homogeneity violations when the number of observations in each group is equal or approximately equal (Vicent, 1999), which is our case. As with the assumption of normality, violation of this assumption does not radically change the F value (Vicent, 1999). We used the post hoc test Games-Howell for the type of data. The classification of the size effect (*i.e.*, measure of the proportion of the total variation in the dependent variable explained by the independent variable) was done according to Pallant (2011). This analysis was performed using the IBM SPSS program (version 19) for a significance level of 5%.

## Results

**Figure 4:**
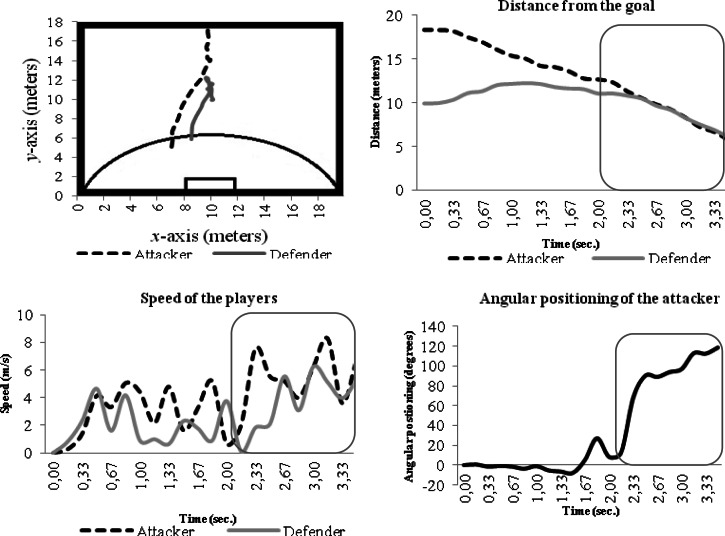
Example of the attacker-defender dyad under the influence of instructional risk constrain

Process variables considered were the distance between players and the goal and the relative speed over time. In the present study, the main goal was to analyze each individual player, thus individual behaviors are provided as an example. Figure 4 shows an example of the player’s behaviour in a specific trial.

The distance between dyad members is fundamental in order to understand the critical point that enables the attacker to overtake its opponent. When the distance between players in relation to the goal decreases, the distance from the attacker-defender dyad also decreases, increasing the players’ speed, especially the attacker’s speed. A gradual increase of the angular positioning (started in 90º) between the attacker-defender dyad was observed until the distance between the attacker and the goal becomes smaller than the distance between the defender and the goal, i.e., when the attacker overtakes the defender at *t* = 2.83 seconds.

Also, it is observed that when the attacker-defender dyad is broken at *t* = 2.83, the angular position between the attacker and the defender significantly increases until it reaches an angle of 118.35º.

**Figure 5:**
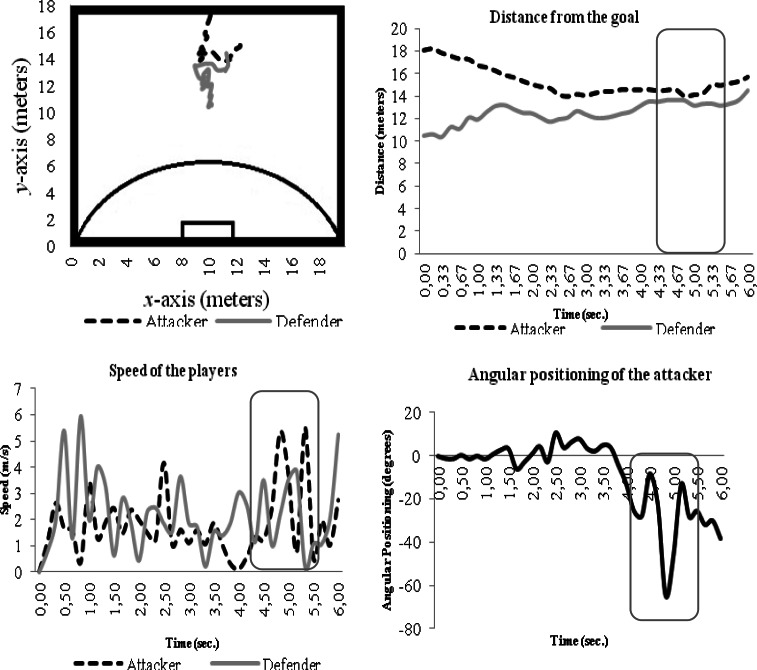
Example of the attacker-defender dyad under the influence of instructional conservative constraint

Although Figure 5 does not present the same characteristics previously depicted, i.e., the attacker does not overtake the defender, it can be verified that when players get closer (almost the same distance between both players and the goal) their speed, especially the attacker’s speed, increases. Similarly to the previous example (Figure 4), the attacker’s highest speed takes place after the defender’s highest speed occurs.

As in the previous example (Figure 5), it is verified that when the attacker is closer to the goal (i.e., *t* = 4.83 seconds), the angular position of the attacker in relation to the defender increases until reaching an angle of 64.42º.

**Figure 6:**
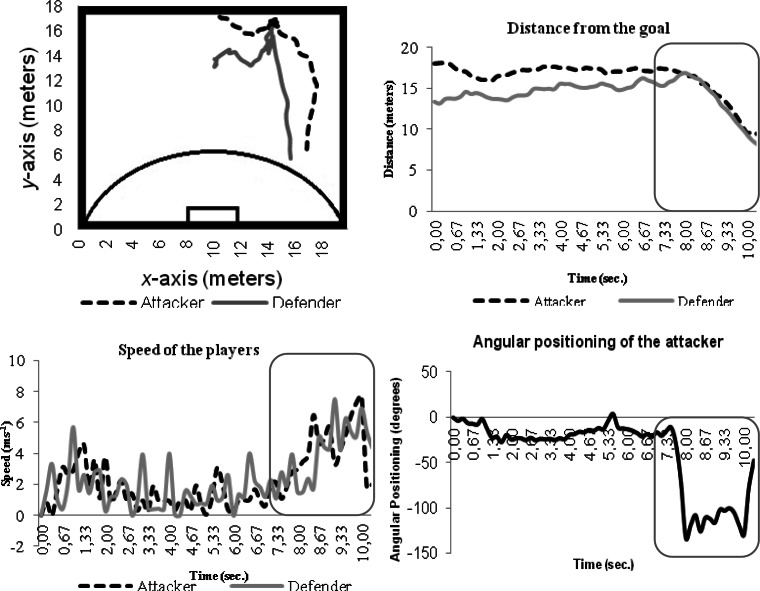
Example of the attacker-defender dyad under the influence of instructional neutral constrain

Figure 6 presents one attempt where the attacker overtakes the defender and the defender recovers his position, stabilizing the dyad. This example demonstrates the importance of speed, distance and angular positioning. It appears that when there is a break in the attacker-defender dyad, the angular positioning of the attacker gradually increases when compared to the defender, followed by a decrease when the defender retrieves his position.

**Figure 7:**
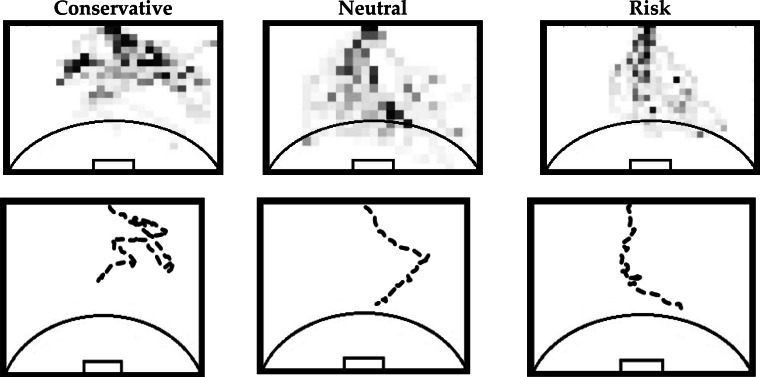
Representative heat maps of 10 trajectories (top) and one random example of these (bottom)

Figure 7 illustrates the attacker’s tendency to go to the left side of the field. However, despite this trend, there is a gradual direction of the player toward the goal as the neutral and risk instructional constraints occur.

A decrease in dispersal areas covered by the attacker was verified, under neutral and risk instructional constraints, being evident the occurrence of directing the ball driving to the goal, i.e., the attacker centralizes his action and trajectory toward the goal. On the other hand, due to the conservative instructional constraint, there is a trajectory of lateralization (i.e., higher occupation of the side zone), thus presenting a greater space dispersion.

The one-way ANOVA showed statistical differences and moderate effect between practice conditions, *i.e.*, instructional constraints (*F*_(2, 327)_ = 30.776; *p* = 0.001; $\eta_{p}^{2}$ = 0.158; *Power* = 1.0). Specifically, the post hoc test, showed differences between the risk constraints and conservative constraints (*p* = 0.001) and neutral constraints (*p* = 0.001). Under the influence of risk constraints, players took less time to complete the offensive attempt when compared to the remaining constraints. Likewise, conservative constraints presented statistically significant differences for the neutral constraints (*p* = 0.035), taking longer to complete the offensive attempt.

## Discussion

The main objective of this study was to analyze the dynamics of the interpersonal dyad formed by the attacker and defender in 1v1 situations with a goalkeeper. Results show that the speed and angular positioning of the attacker are key factors when trying to unbalance the attacker-defender dyad. Thus, when the interpersonal distance decreases, there is an increase in players’ speed and angular positioning, especially by the attacker. For instance, the speed increase is used to break the stability and balance of the opponent while the increase of the angular positioning in relation to the defender means that the attacker tries to overtake the defender on one side, in order to avoid the proximity of the ball with the defenders’ feet.

It is also confirmed that the interpersonal distance is closely related to players’ speed. These features seem to be crucial to attacker’s action to invert the balance of the defender. These statements are consistent with the research of Duarte et al. (2010).

The analysis of the angular variation between the attacker and the defender presents a transition phase (i.e., the disruption of the attacker-defender dyad) identified by an increase of the attacker’s angular variation. Thus, in order to overtake the defender, the attacker must increase their absolute angular positioning.

The attacker-defender dyad’s behaviour, emerged from the constant exploration of the environment (e.g., defender, ball position, goal position) where the attacker tries to explore the best options during the action, confirming the study of Passos et al. (2006). This statement can be supported by the regular changes of the attacker’s actions during the trials, meaning that he did not have a predefined plan, but yet the behaviour emerges from the specific context. In addition, like in the work of Davis et al. (2006), the player’s action emerges from the perception-action cycles where the player tries to act in order to collect new information, thus improving the opportunity to achieve the main goal. Furthermore, like the study of Duarte et al. (2012), the player’s action in cooperation-opposition games and their subphases (e.g., 1x1, 2x2) can be characterized as a self-held process that depends on constant interactions between dyad members, thus trying to achieve the main goals, breaking the defensive balance of opponents.

Thus, there is strong evidence to affirm that the player’s action is not predetermined, but emerges from a set of perceptions that arise from environment interactions (Clemente et al., 2012). Also, the variability of attacker’s actions is a permanent source of new perception-action cycles, and thus the behaviour cannot be previously determined, but emerges from the contextual information.

About the influence of instructional constraints in risk situations, players’ behaviour is distinguished since a significant reduction in the variability of the attacker’ trajectories are observed through the heat maps. Under risk information constraints, the completion time is statistically significantly shorter when compared to others information constraints, as it is possible to observe in the post-hoc values.

When the instruction to keep the ball was given, unlike the risk constraint, there was a lateralization of the action (as it is possible to observe through the heat maps), increasing the variability and dispersion of the attacker’s trajectories. Also, it a statistically significant time increase to complete the offensive attempt was also found when compared to the other instruction constraints (considering the post hoc tests). The attacker possibly exploits all the available space to maximize possession time.

## Conclusions

The main objective of this study was to analyze the dynamics of the interpersonal dyad formed by the attacker and the defender in 1v1 situations with a goalkeeper. Additionally, this paper aimed to analyze the influence of different instructional constraints on players’ performance. Results show that the speed and angular positioning of the attacker are key factors when trying to unbalance the attacker-defender dyad in all conditions of practice. The task constraints proved to be essential to control the spatial distribution of players as well as the time to complete the offensive attempt, as it is possible to observe through the heat maps of players’ trajectories and the statistical analysis of the time spent in each instructional constraint. It was also found that the spatial distribution of the attacker varies depending on the given instruction. Based on the obtained data, heat maps are particularly useful to analyze athletes’ trajectories trends. Finally, performing the transfer of this research for sports training, we can conclude that the coach may provide several constraints in order to guide players, thus allowing them to search for new solutions that may be adjustable to individual variability and unpredictability of the football game (Davids et al., 2005).

## Figures and Tables

**Figure 2:**
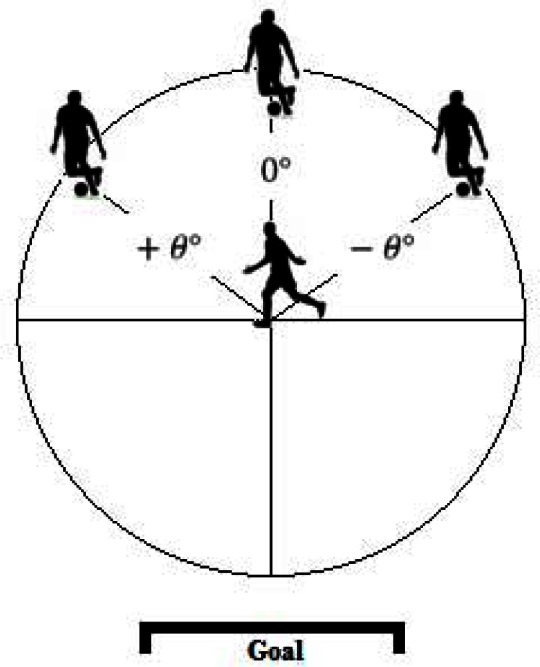
Angular Positioning Between Players
